# Role of CCR7 on dendritic cell-mediated immune tolerance in the airways of allergy-induced asthmatic rats

**DOI:** 10.3892/mmr.2019.10694

**Published:** 2019-09-20

**Authors:** Yi Li, Yongcheng Du, Aizhen Zhang, Rui Jiang, Xin Nie, Xue Xiong

**Affiliations:** Department of Respiration Medicine, People's Hospital of Shanxi Province, Taiyuan, Shanxi 030001, P.R. China

**Keywords:** CC chemokine receptor 7, dendritic cells, allergic asthma, allergy-induced asthma, immune tolerance

## Abstract

Dendritic cells (DCs) have an important role in initiating and maintaining the immune inflammatory response in allergic asthma, and CC chemokine receptor 7 (CCR7) is directly involved in the pathogenesis of DC- and T cell-mediated allergic asthma. The present study aimed to investigate the effects of CCR7 on DC-mediated immune tolerance in allergic asthma. In the present study, bone marrow-derived DCs were transfected with an adenovirus encoding the rat CCR7 gene or a short hairpin RNA targeting CCR7 (sh-CCR7). Rats injected with DCs overexpressing CCR7 or presenting CCR7 knockdown were examined. After the rats were injected with DCs via the tail vein, bronchoalveolar lavage fluid was collected to assess its cellular composition. The protein expression levels of CCR7 in DCs were determined using immunohistochemistry and western blot analysis. The protein expression levels of interferon-γ (IFN-γ), interleukin-4 (IL-4), IL-10, IL-12, transforming growth factor-β (TGF-β) and immunoglobulin E (IgE) were determined by ELISA. Compared with the control group, the protein expression level of CCR7 was significantly higher in the CCR7 overexpression group and significantly lower in sh-CCR7 group. Similarly, the number of DCs was higher in the CCR7 overexpression group and lower in the sh-CCR7 group. The protein expression levels of IL-10 and TGF-β were significantly lower in the CCR7 overexpression group and higher in the sh-CCR7 group. In addition, the expression levels of IL-4, IL-12, IFN-γ and IgE were higher in the CCR7 overexpression group and lower in the sh-CCR7 group. The present results suggested that the role of cytokines and IgE in immune inflammation and immune tolerance in allergic asthma may be associated with the expression level of CCR7 in DCs, suggesting that CCR7 may serve a role in DC-mediated immune tolerance in allergic asthma.

## Introduction

Bronchial asthma is a chronic airway inflammatory disease. As of 2008, ~300 million people worldwide have been diagnosed with asthma, with China accounting for ~10% (~30 million) of all cases (1). As a potential life-threating condition, asthma has become a serious health problem worldwide. Recently established treatment protocols have significantly improved asthma control rates; however, symptomatic treatment represents the primary available therapeutic option, and inhaled corticosteroids and bronchodilators are the most common medications used to treat asthma symptoms (2). Currently, the absence of effective etiological treatments represents a limitation in improving the asthma control rate.

Allergic asthma is a common type of asthma, and children are twice as likely to develop this disease compared with adults (3). Immune-mediated inflammation is the principal factor involved in allergic asthma pathogenesis, and a variety of inflammatory mediators and cytokines are involved in this process, including interferon-γ (IFN-γ), interleukin-4 (IL-4) and immunoglobulin E (IgE) (4). Dendritic cells (DCs) are involved in the initiation and maintenance of the inflammatory chain reaction underlying allergic asthma (5,6). Previous studies have shown that DC surface molecules, including major histocompatibility complex class II (MHC-II) molecules and costimulatory proteins (7,8), and DC-secreted cytokines (9) can regulate the differentiation of naïve T cells into type 1 or type 2 T-helper (Th2) cells, or regulatory T cells (Tregs). DCs are associated with airway epithelial cells, mast cells and eosinophils, three types of cells that are involved in asthmatic pathology (10–12). Asthma is caused by the activation and recruitment of inflammatory cells and secretion of pro-inflammatory factors following exposure to an antigen (13), suggesting that asthma could be treated by suppressing this process.

CC chemokine receptor 7 (CCR7) is primarily expressed on the surface of DCs, T-lymphocytes and B-lymphocytes (14) and it has been shown to promote the internalization of antigens by DCs, and to regulate cell survival, migration, and to induce DC maturation (15,16). Immune tolerance is the state of unresponsiveness of the immune system to a particular antigen (17). A previous study has shown that the CCR7-dependent migration of DCs from the lungs to draining lymph nodes is involved in the transport of inhaled silver particles, and this process is essential to induce peripheral tolerance of T cells (18). However, this previous study was primarily focused on the process of immune tolerance associated with antigen presentation. The mechanism underlying the role of CCR7-expressing DCs in the regulation of immune tolerance in the airways during allergic asthma remains unclear and requires further investigation. Therefore, the present study investigated the effects of CCR7 knockdown and overexpression on DC-mediated immune tolerance in the lungs of rats with allergic asthma.

## Materials and methods

### 

#### Culture of bone marrow-derived immature DCs (imDCs)

The present study was approved by The Ethics Committees of The People's Hospital of Shanxi Province. A total of 33 specific pathogen-free (SPF) male Wistar rats (age, 6–8 weeks; weight, 180–200 g) were obtained from The Laboratory Animal Center of Hangzhou Hibio Technology Co., Ltd. The animals were acclimatized to laboratory conditions (23°C, 12-h light/dark cycle, 50% humidity and *ad libitum* access to food and water) for 2 weeks prior to experimentation. Animals were sacrificed by intravenous injection of pentobarbital sodium (150 mg/kg) and animal death was confirmed by lack of reflexes, heartbeat and breathing. Specific tissues were collected for experimentation. Bone marrow was collected from the femurs and tibiae of Wistar rats as previously described (19). The intact femurs and tibiae were kept in 70% ethanol for 30 min, and then rinsed with fresh RPMI medium (Gibco; Thermo Fisher Scientific, Inc.). The extremities of the femurs and tibiae were removed with scissors, and the bone marrow was flushed using 5–10 ml medium with a 19-gauge syringe. A lysis buffer containing ammonium chloride (KHCO_3_ 1.0 g/l; NH_4_CL 8.3 g/l; EDTA-Na_2_ 0.037 g/l) was used to lyse red blood cells and the samples were washed twice to isolate the bone marrow cells. Bone marrow cells were resuspended in RPMI medium supplemented with recombinant rat IL-4 (10 ng/ml; PeproTech, Inc.) and recombinant rat granulocyte monocyte colony-stimulating factor (GM-CSF; 10 ng/ml; PeproTech, Inc.), and cultured at a density of 1×10^6^ cells/ml in six-well plates with 3 ml culture medium/well at 37°C in a 5% CO_2_ humidified incubator. Fresh culture medium and cytokines were added at day 3. Cell aggregates attached to the dish surface were observed between day 3 and 4. The culture medium was removed and replaced by fresh medium with GM-CSF at day 5 and 6.

The phenotype of the cultured dendritic cells was examined by investigating the protein expression levels of α E2 integrin (OX62) and MHC-II, as assessed by flow cytometry (Fig. S1), conducted as follows: One well of a 6-well plate was centrifuged at 300 × g for 5 min at 37°C, and the cell pellet was collected and resuspended in 5X volume of washing solution (0.01 M PBS). The cells were rinsed twice and resuspended in 200 µl of staining buffer (cat. no. 420201; BioLegend, Inc.) at a concentration of 1×10^6^ cells/ml. The cells were equally divided into two 1.5-ml centrifuge tubes; one tube was an experimental group, whereas the other was a blank group. Phycoerythrin (PE)-conjugated anti-MHC class II (1:50; cat. no. 12-0920-82; Invitrogen; Thermo Fisher Scientific, Inc.) and PE-conjugated anti-Ox62 (1:50; cat. no. 12-1030-82; Invitrogen; Thermo Fisher Scientific, Inc.) antibodies were added separately and incubated for 30 min at room temperature in the dark; the blank group for each assay was incubated without primary antibody. MHC-II and OX62 were directly detected using an Accuri C6 flow cytometer (BD Biosciences); the blank group was used to determine the negative region. The test was repeated three times.

#### Transfection of imDCs with short hairpin RNA (shRNA) targeting CCR7

The sequences of the shRNA targeting CCR7 and the control sequences were as follows: shRNA-CCR7, 5′-TGGATCTTTGGTGCCTACCTGTGTA-3′; control shRNA, 5′-TTCTCCGAACGTGTCACGTAA-3′. shRNA-CCR7 and control shRNA was inserted into a pHBAd-U6-GFP backbone, which was packaged into an adenovirus. The 293A cells were transfected with the shCCR7 plasmid, and the adenovirus was harvested. The plasmid, shRNA and adenovirus were all supplied by Hangzhou Hibio Technology Co., Ltd. As control, an empty vector was used, and the infection efficiency was assessed by western blot analysis (Fig. S2). Then, 1×10^6^ imDCs were suspended in 1 ml adenoviral supernatant (MOI=50) with 1% FBS (Invitrogen; Thermo Fisher Scientific, Inc.), 10 ng/ml GM-CSF and 10 ng/ml IL-4 at 37°C for 72 h. Subsequently, cells were centrifuged at 300 × g at 37°C for 2 h. After infection, imDCs were washed twice in PBS and incubated with RPMI-1640 medium containing 10% fetal calf serum (FCS; Gibco; Thermo Fisher Scientific, Inc.) supplemented with 10 ng/ml GM-CSF and 10 ng/ml IL-4 at 37°C with 5% CO_2_. The cells were harvested for injection 48 h after infection.

#### Transfection of imDC with adenovirus overexpressing CCR7

*Not*I and *Pst*I restriction sites were added to the ends of the CCR7 coding sequence, which was cloned into a pDC316-MCMV-ZsGreen backbone, which was packaged into an adenovirus. The recombinant adenoviral vector containing the rat *CCR7* gene was prepared as previously described by Hibio Technology Co., Ltd. (20), and the centrifugal enhancement method was used to increase the infection efficiency (21). Empty plasmid vector was used as negative control. Viral packaging was verified by the detection of green fluorescent protein via fluorescence microscopy (data not shown). Subsequently, 1×10^6^ imDCs were suspended with 1% FBS, 10 ng/ml GM-CSF and 10 ng/ml IL-4, adding the adenovirus at MOI=50, and cells were subsequently centrifuged at 300 × g at 37°C for 2 h. After infection, the imDCs were washed twice in PBS and incubated with RPMI-1640 medium containing 10% FCS supplemented with 10 ng/ml GM-CSF and 10 ng/ml IL-4 at 37°C with 5% CO_2_. The cells were harvested for injection 48 h after transfection. The infection efficiency of CCR7 was determined by western blot analysis (Fig. S3).

#### Establishment of animal models

Healthy SPF male Wistar rats were used to establish an animal model of allergic asthma, according to a modified previously described protocol (22). Rats were immunized by intraperitoneal injection of 100 µg ovalbumin (OVA; Sigma-Aldrich; Merck KGaA) adsorbed into 400 µg aluminium hydroxide in 0.2 ml sterile saline (0.9%) on day 1 and 8. After 2 weeks, rats were exposed to aerosol spray containing 1% OVA in 0.9% sterile saline for 30 min every day, 6 days every week, for a total of 8 weeks. The experimental rats were divided into three groups (n=10 in each group) as follows: i) Control (Con) group, rats injected with 2×10^5^ cultured wild-type imDCs; ii) CCR7 overexpression (Over) group, rats injected with 2×10^5^ cultured imDCs infected with adenoviral particles overexpressing CCR7; and iii) CCR7 interference (Sh) group, containing rats injected with 2×10^5^ cultured imDCs infected with adenoviral particles encoding a shRNA targeting CCR7. imDCs were injected via the tail vein at day 1, 10 and 18.

#### Immunohistochemistry of CCR7 and OX62

For immunohistochemical staining, lung tissues were fixed at room temperature for 5 days using 4% formaldehyde solution, embedded in paraffin and sectioned (4-µm), dewaxed in 100% xylene (twice in total, 20 min each time), rehydrated in a graded alcohol series (100% for 5 min, 95% for 5 min, 80% for 5 min), and incubated with 0.3% H_2_O_2_ in methanol to block the endogenous peroxidase activity for 15 min at 37°C. Mounted sections were boiled in 10 mM citrate solution (pH 6.0) for 20 min for antigen retrieval, and incubated overnight at 4°C with the following primary antibodies: Anti-rat CCR7 (1:50; cat. no. bs-1305R; Bioss, Inc.) and anti-rat OX62 (1:50; cat. no. sc-53085; Santa Cruz Biotechnology, Inc.). Horseradish peroxidase-conjugated secondary antibody (1:25; cat. no. PV-6001; Beijing Zhongshan Golen Bridge Biotechnology Co., Ltd.; OriGene Technologies, Inc.) was incubated with the sections at 37°C for 30 min. A two-step technique (SuperPicture Third Generation IHC Detection kit; Invitrogen; Thermo Fisher Scientific, Inc.) was used to visualize the stained samples, and 0.1 mg/ml 3,3′-diaminobenzidine diluted in a 0.02% H_2_O_2_ solution (Vector Laboratories, Inc.) was used as chromogen for 2 min at room temperature. The tissue sections were counterstained with hematoxylin (1 g/ml, 2 min) and eosin (5 g/l, 30 sec) at room temperature, and observed using a light microscope (magnifications, ×100 and ×400; Olympus Corporation).

#### Western blot assay

Lung tissues were homogenized in RIPA buffer (Pierce; Thermo Fisher Scientific, Inc.) and the protein concentration was determined using the bicinchoninic acid protein assay (Pierce; Thermo Fisher Scientific, Inc.). Total protein was separated by SDS-PAGE on 10% gels. In total, 30 µg protein was loaded in each lane. Proteins were then transferred to PVDF membranes (EMD Millipore). Membranes were blocked for 2 h at room temperature in 5% non-fat dry milk and incubated with a primary antibody anti-rat CCR7 (1:1,000; cat. no. ab32527; Abcam) or anti-OX62 (1:1,000; cat. no. bs-1274R; Bioss, Inc.) at 4°C overnight. Membranes were then incubated with the appropriate horseradish peroxidase-labeled anti-mouse [1:1,000; cat. no. GAM007; MultiSciences (Lianke) Biotech Co., Ltd.] or anti-rabbit IgG [1:1,000; cat. no. GAR0072; MultiSciences (Lianke) Biotech Co., Ltd.] secondary antibodies at room temperature for 50 min. Anti-β-actin (1:1,000; cat. no. sc-47778; Santa Cruz Biotechnology Inc.) or GAPDH (1:1,000; cat. no. sc-32233; Santa Cruz Biotechnology Inc.) was used as the loading control. Bands were visualized by autoradiography (ChemiDoc XR+System; Bio-Rad Laboratories, Inc.) and quantified by densitometry (ImageJ V1.46; National Institutes of Health). The results were normalized to GAPDH or to β-actin. All experiments were performed in triplicate.

#### Bronchoalveolar lavage fluid (BALF) collection and cell counting

After the animal model was successfully established (8 weeks), the animals were anesthetized and the lungs were exposed. The right principal bronchus was occluded using a hemostat clamp. A total of 4 ml saline was used for the bronchoalveolar lavage in the left lung, and 3–4 ml of fluid was collected after flushing twice. The alveolar lavage fluid was centrifuged at 300 × g for 10 min at room temperature, and the supernatant was used for ELISA. Next, 0.5 ml saline was added to 0.5 ml BALF containing sediment and mixed. Then, the samples were smeared and the cells were counted using the Wright-Giemsa staining technique [Wright's dyeing powder (1 g), Jimsa dyeing powder (0.5 g), 20 min incubation, room temperature] and observed with a CX21 light microscope (Olympus Corporation). Cells were counted under a microscope using a hemocytometer and a counting place (25×16 grid cells); the number of cells in each corner of the plate was calculated.

#### ELISA

After the animal model was successfully established, blood (4 ml) was extracted from the vena cava, anticoagulated with heparin and centrifuged (500 × g for 10 min at room temperature), and serum was collected. ELISA kits were used for measuring IFN-γ (cat. no. EK0374), IL-4 (cat. no. EK0406), IL-12 (cat. no. A01152-2), IL-10 (cat. no. EK0418), TGF-β (cat. no. EK0514; all Boster Biological Technology) and IgE (cat. no. E-EL-R0517c; Elabscience Biotechnology, Inc.) concentrations in serum and BALF supernatant, according to the manufacturer's protocol.

#### Statistical analysis

All assays were repeated three times. The data are presented as the mean ± SD. Statistical analysis was performed using SPSS 11.5 software (SPSS, Inc.). One-way ANOVA was performed to compare multiple groups, followed by Student-Newman-Keuls-Q post-hoc test. P<0.05 was considered to indicate a statistically significant difference.

## Results

### 

#### Expression of CCR7 in lung tissues following CCR7-overexpressing DC injection in vivo

**Figure 1.**
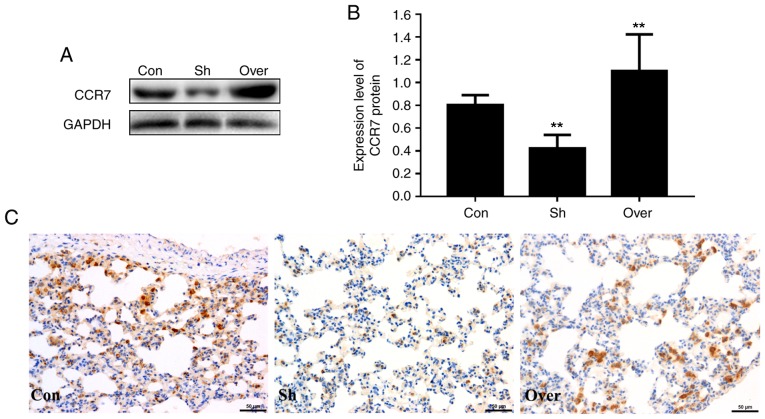
Expression of CCR7 in lung tissue. (A) CCR7 protein expression levels in the three experimental groups were assessed using western blot analysis. GAPDH was used as loading control. (B) Densitometric analysis of the western blot. (C) CCR7 expression examined by immunohistochemical analysis in the airway of rats. Magnification, ×100. **P<0.01 vs. Con. Con, control group; Over, CCR7 overexpression group; Sh, CCR7 knockdown group; CCR7, CC chemokine receptor 7.

Immunohistochemical analysis suggested that the protein expression level of CCR7 was increased in the airways of rats in the CCR7-overexpressing group (Fig. 1). In addition, the cell membranes were stained yellow and brown, indicating the localization of CCR7 in the peribronchial stroma (Fig. 1C). Compared with the control group, the protein expression of CCR7 was significantly higher in the airways of rats in the overexpression adenovirus-infected (Over) group and lower in the shRNA adenovirus-infected (Sh) group (P<0.01; Fig. 1B). The protein expression levels of CCR7 in various groups detected by western blotting was consistent with the immunohistochemical results (Fig. 1A).

#### CCR7-overexpressing DCs promote inflammatory cell infiltration in the lungs of rats with allergic asthma

**Figure 2.**
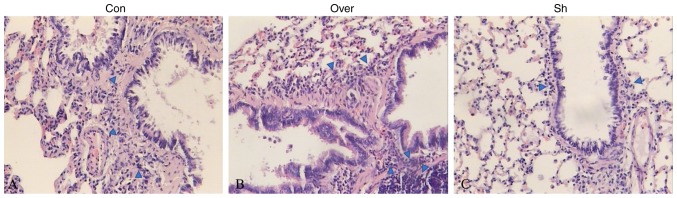
Pathological examination of lung tissues as assessed by hematoxylin and eosin staining. Inflammatory cells were labeled with blue arrows. (A) Con group: Smooth muscle cell proliferation and inflammatory cell infiltration. (B) Over group: Increased number of infiltrating inflammatory cells in the airway and lung tissue, and the airway wall was markedly thicker compared with the Con group. (C) Sh group: Bronchiole structure was normal, and a small number of infiltrating inflammatory cell was observed in the lung tissue. Magnification, ×100. CCR7, CC chemokine receptor 7; Con, control group; Over, CC chemokine receptor 7 overexpression group; Sh, CCR7 knockdown group.

HE staining suggested the presence of numerous inflammatory cells in the lung tissues in the control group (Fig. 2). Additionally, the number of infiltrated inflammatory cells, including lymphocytes, eosinophils and neutrophils (as determined by cell morphology following H&E staining), was increased in the airway and lung tissues in the Over group compared with the Con group, and the airway wall was markedly thicker in the CCR7 overexpression group (Fig. S4). In the Sh group, the bronchiole structure was normal and the number of inflammatory cells infiltrating the lung tissue was reduced.

#### Expression of DC-specific antigens in lung tissues is positively associated with the expression level of CCR7

**Figure 3.**
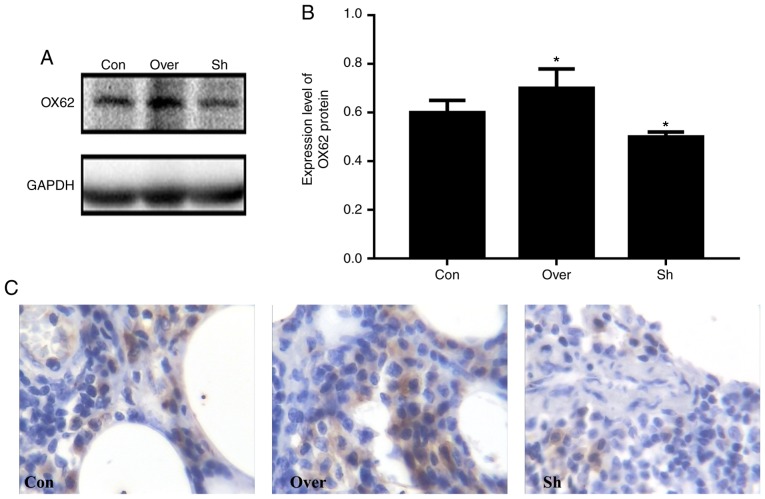
Increased expression of OX62 in lung tissue. (A) OX62 expression was assessed using western blot analysis. GAPDH was used as loading control. (B) Densitometric analysis. (C) OX62 expression examined by immunohistochemical analysis in the rat airways. Magnification, ×400. *P<0.05 vs. Con. OX62, α E2 integrin; CCR7, CC chemokine receptor 7; Con, control group; Over, CCR7 overexpression group; Sh, CCR7 knockdown group.

OX62 was used a marker for DC detection in the airways as it is specifically expressed by DCs (23). The CCR7 Over group exhibited higher expression levels of OX62 compared with the Con and Sh groups (Fig. 3). In addition, the protein expression level of OX62 was lower in the CCR7 interference group compared with the control group (P<0.05).

#### Positive correlation between the expression levels of CCR7 and the presence of leukocytes, neutrophils and lymphocytes in BALF

**Figure 4.**
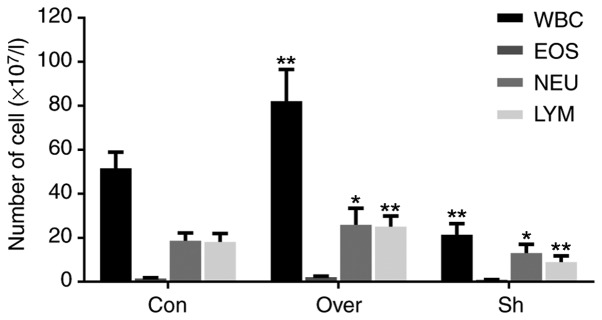
Cell count and classification of immune cells in bronchoalveolar lavage fluid. Numbers of WBC, NEU, EOS and LYM in each group. *P<0.05 vs. Con; **P<0.01 vs. Con. CCR7, CC chemokine receptor 7; Con, control group; Over, CCR7 overexpression group; Sh, CCR7 knockdown group; WBC, leukocytes; EOS, eosinophil; NEU, neutrophils; LYM, lymphocytes.

The numbers of leukocytes (P<0.01), neutrophils (P<0.05) and lymphocytes (P<0.01) were higher in the CCR7 Over group compared with the Con group and the Sh group (Fig. 4). Conversely, the expression of eosinophils was not affected by CCR7 overexpression. The present data suggested that CCR7 expression could affect the number and types of immune cells recruited to the airways and infiltrating the lung tissues.

#### Expression levels of cytokines and IgE in the BALF and serum are associated with the expression level of CCR7

**Figure 5.**
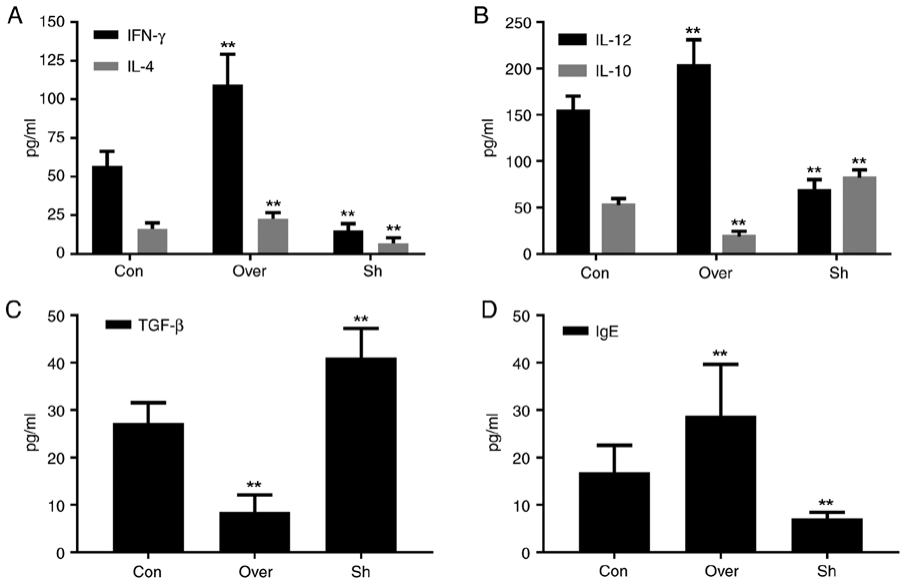
Protein expression levels of (A) IFN-γ and IL-4, (B) IL-12 and IL-10, (C) TGF-β and (D) IgE in bronchoalveolar lavage fluid. Protein expression levels of various cytokines were assessed by ELISA. **P<0.01 vs. Con. CCR7, CC chemokine receptor 7; IFN-γ, interferon-γ; IL, interleukin; Con, control group; Over, CCR7 overexpression group; Sh, CCR7 knockdown group; TGF-β, transforming growth factor-β; IgE, immunoglobulin E.

**Figure 6.**
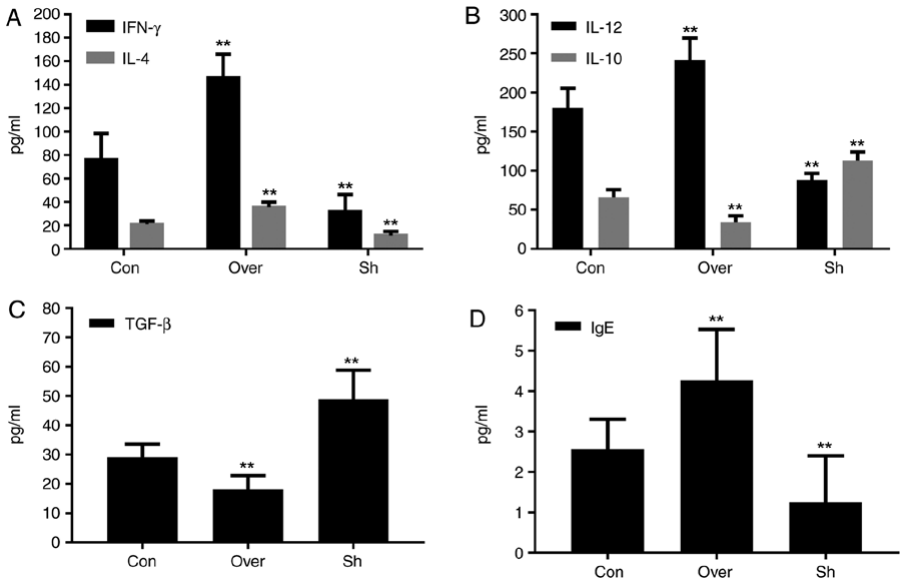
Protein expressions of (A) IFN-γ and IL-4, (B) IL-12 and IL-10, (C) TGF-β and (D) IgE in serum. Protein expression levels of various cytokines were assessed by ELISA. **P<0.01 vs. control group. CCR7, CC chemokine receptor 7; IFN-γ, interferon-γ; IL, interleukin; Con, control group; Over, CCR7 overexpression group; Sh, CCR7 knockdown group; TGF-β, transforming growth factor-β; IgE, immunoglobulin E.

Compared with the control group, the protein expression levels of IL-12, IL-4, IFN-γ and IgE, in both the BALF supernatant (Fig. 5) and serum (Fig. 6), were increased in the CCR7 Over group compared with the Con and the Sh groups (P<0.01). Compared with the control group, the protein expression levels of IL-10 and TGF-β in the BALF supernatant (Fig. 5) and serum (Fig. 6) were significantly lower in the CCR7 Over group, and higher in the Sh group (P<0.01).

## Discussion

One of the most important functions of the immune system is to prevent pathogen-associated damage, which is limited through effective recognition of the exogenous antigens and initiation of the immune response (24). The immune system allows the body to maintain its internal environment via the immune tolerance mechanism (25). Both immune response and immune tolerance mechanisms serve important roles in allergy-induced asthma (26). Therefore, in the present study, the effects of CCR7 overexpression and knockdown in DCs on the mechanisms of immune tolerance were investigated in an animal model of allergic asthma.

In the present study, DC markers were examined, and the expression levels of OX62 were decreased following shRNA-mediated CCR7 knockdown in DCs. The present results suggested that CCR7 may serve an important role in the regulation of the immune response in DC-induced asthma. Previous studies have demonstrated that imDCs can internalize, process and deliver antigens, and it is also associated with the induction of immune tolerance (9); however, mature DCs exhibit immune stimulating abilities (9). Interestingly, CCR7 expression is limited to mature DCs (27). imDCs have been used as a model to study the association between changes in the expression level of cytokines and the immune inflammatory response in allergic asthma (28). In the present study, HE and Wright-Giemsa staining of lung tissues and BALF showed an increased number of infiltrating inflammatory cell in the CCR7 overexpression group. The present findings suggested that CCR7 may promote immune inflammation. Conversely, there was no significant association between eosinophils and CCR7 expression; the increase and aggregation of eosinophils may be related to other factors, such as IL-5 (29). However, a previous study showed that CCR7-deficient animals fail to induce tolerance to inhaled environmental innocuous antigens (17). The reasons underlying the differences between the present and this previous study may be multifactorial. Notably, the animal model is different; in the present study, continuous inhalation of OVA was used to induce bronchial asthma in rats, whereas the previous study established an immune tolerance model via the inhalation of increasing doses of the antigens. Moreover, the present study used a rat allergy model, whereas the previous study was performed in mice. In addition, the CCR7-deficient animals in the previous study may exhibit additional defects, such as impaired T cell recirculation, that may influence the immune system in these animals.

CCR7 possesses a variety of functions and properties in DCs. A previous study has shown that knockdown of CCR7 increases the expression levels of CD80, CD86, IFN-γ, IL-12/23 and IL-1α in DC (30). CCR7 silencing also increases the resistance to infection by increasing the number of neutrophils in the lung airways (31). In the present study, various cytokines, including IgE, were found to be involved in allergy-induced asthma and their expression level decreased following knockdown of CCR7. The present results suggested that a decrease in the expression level of CCR7 may suppress the immune response in patients with allergy-induced asthma. Accumulating evidence demonstrated that the mucosa in asthmatic airways contains a large number of activated Th cells, as well as higher levels of IL-4 and IL-5. *In vitro* studies have shown that IL-4 can induce B lymphocytes to synthesize IgE, promoting airway hyper-responsiveness (32–34), although IFN-γ inhibits this effect (35). IL-12 is a major cytokine that regulates immune balance by promoting the expression of certain cytokines, such as IFN-γ, and inhibiting the secretion of other cytokines, such as IL-4 and IL-5 (36). Therefore, IFN-γ, IL-4, IL-12 and IgE serve important roles in the pathogenesis of allergy-induced asthma. Overall, decreased CCR7 expression not only reduced inflammatory cell infiltration, but also decreased the expression levels of various inflammatory factors.

IL-10 and TGF-β are cytokines involved in the process of immune tolerance (37,38). IL-10 exhibits a wide range of functions, including the inhibition of Th2 cytokine and IgE production and it is involved in decreasing mast cell and eosinophil function (39). In addition, IL-10 increases the secretion of IgG4 and regulates the IgG4/IgE ratio (40). TGF-β is a pro-inflammatory cytokine that regulates lymphocyte homeostasis, suppresses Th2 cell activation and promotes Treg cell differentiation (41). In the present study, CCR7 knockdown increased the protein expression levels of IL-10 and TGF-β in allergy-induced asthma, suggesting that CCR7 may serve an important role in immune tolerance in allergy-induced asthma. The induction of T cell antigen-specific immune tolerance may represent a novel strategy for the treatment of various immune inflammatory diseases, including allergy-induced asthma.

The immune tolerance to allergens in asthmatic patients is due to the activation and proliferation of Th cells (26). Therefore, mechanisms underlying immune tolerance defects may be important for the pathogenesis of allergy-induced asthma. Knockdown of CCR7 in DCs caused the cells to remain in an immature state, promoting immune tolerance. This effect may further reduce the activity of DCs, leading to decreases in the expression levels of cytokines involved in the immune response in allergy-induced asthma, and increases in the expression levels of cytokines involved in immune tolerance.

CCR7 has important roles in DC-mediated immune inflammation and immune tolerance in patients with allergy-induced asthma (17). Notably, the present study presents certain limitations, since the expression level of CCR7 in DCs was investigated only in the lungs. In the future, it may be useful to examine the role of CCR7-expressing DCs in other tissues prone to allergic inflammation. Moreover, further studies are required to examine the CCR7-dependent chemotaxis and CCR7-mediated signal transduction pathways, which may provide insights into novel therapeutic approaches for the treatment of patients with allergic asthma.

## Supplementary Material

Supporting Data

## Data Availability

The datasets used and/or analyzed during the present study are available from the corresponding author on reasonable request.
