# Supplementary material for: Role of CCR7 on dendritic cell-mediated immune tolerance in the airways of allergy-induced asthmatic rats
Source: Mol Med Rep. 2019 Sep 20;20(5):4425–32. doi: 10.3892/mmr.2019.10694 (PMC6797982; doi:10.3892/mmr.2019.10694)

Figure S1. Phenotype of cultured dendritic cells assessed by OX62 and MHC-II flow cytometric assays. Black lines represent blank groups; red lines represent the different detection indexes. OX62, α E2 integrin; MHC-II, major histocompatibility complex class II.

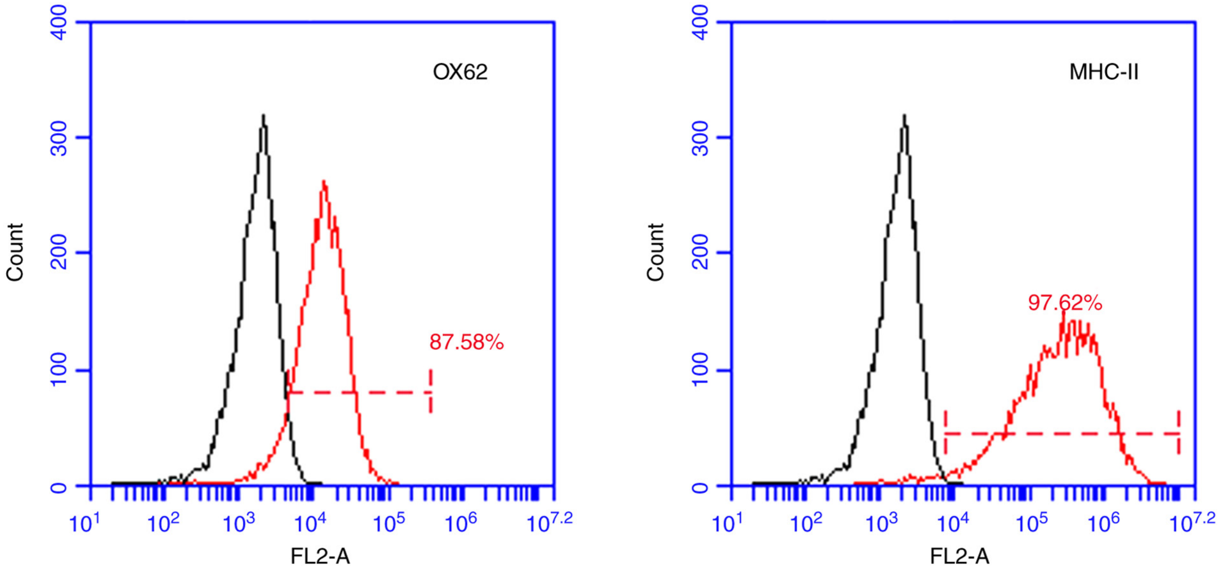

Figure S2. Infection efficiency of a vector encoding a small interfering RNA targeting CCR7. imDCs were infected with an adenoviral plasmid carrying a siRNA targeting CCR7. Efficient knockdown was assessed by western blot analysis. CCR7, CC chemokine receptor 7; CK, blank control group; NC, empty vector; Sh, CCR7 knockdown group; imDCs, immature dendritic cell.

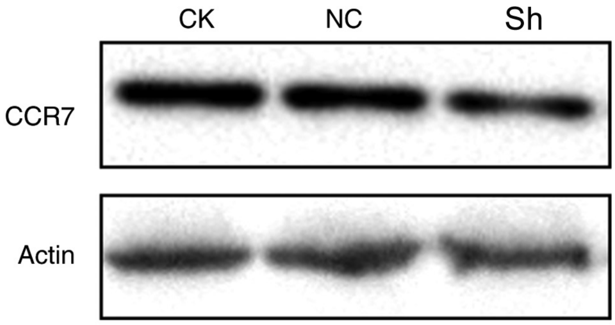

Figure S3. Infection efficiency of a vector overexpressing CCR7. imDCs were infected with a viral vector overexpressing CCR7 gene, and the infection efficiency was assessed by western blot analysis. CK, blank control group; NC, empty vector; Over, CCR7 overexpression group; CCR7, CC chemokine receptor 7.

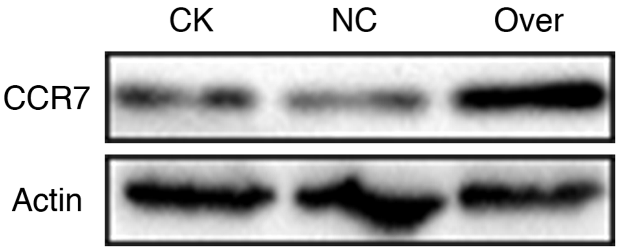

Figure S4. Pathology of lung tissues assessed by hematoxylin and eosin staining. Lymphocytes and eosinophils were the primary infiltrating inflammatory cells in the airways of asthmatic rats in the Con group. In the Over group, an increased number of infiltrating lymphocytes, eosinophils and neutrophils was identified in the airway and lung tissue. In the Sh group, the bronchiole structure was normal, a reduced number of infiltrating inflammatory cells, such as eosinophils and neutrophils, was observed in the lung tissues. Magnification, x400. Lymphocytes are indicated by yellow arrows. Eosinophils are indicated by blue arrows. Neutrophils are indicated by green arrows. CCR7, CC chemokine receptor 7; Con, control group; Over, CCR7 overexpression group; Sh, CCR7 knockdown group.

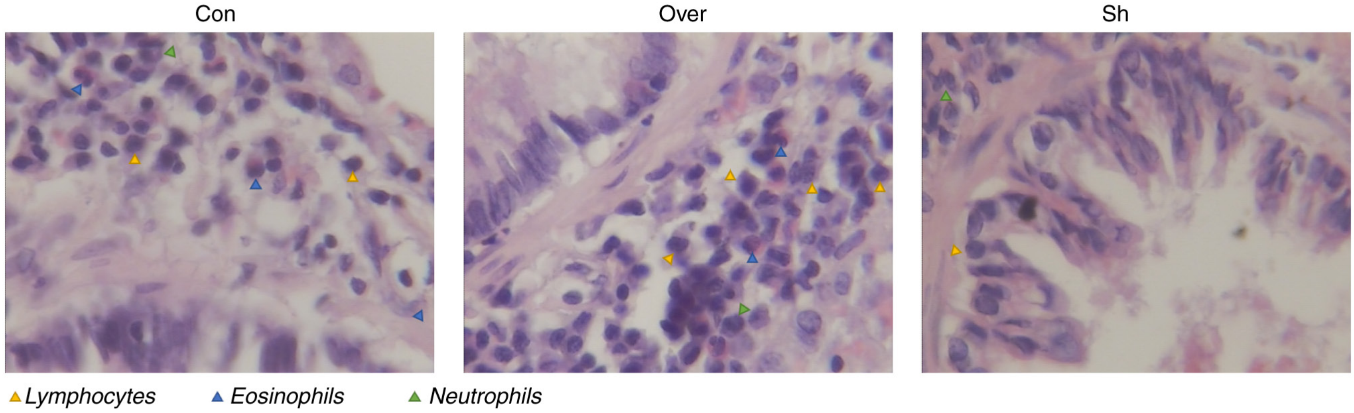

Supplement: Supporting Data [file Supplementary_Data.pdf]
